# Feasibility and Acceptability of a Smoking Cessation Program for Individuals Released From an Urban, Pretrial Jail: A Pilot Randomized Clinical Trial

**DOI:** 10.1001/jamanetworkopen.2021.15687

**Published:** 2021-07-06

**Authors:** Tyler N. A. Winkelman, Becky R. Ford, Shira Dunsiger, Michelle Chrastek, Sarah Cameron, Ella Strother, Beth C. Bock, Andrew M. Busch

**Affiliations:** 1 Health, Homelessness, and Criminal Justice Lab, Hennepin Healthcare Research Institute, Minneapolis, Minnesota; 2 Division of General Internal Medicine, Department of Medicine, Hennepin Healthcare, Minneapolis, Minnesota; 3 Center for Health Promotion and Health Equity, Department of Behavioral and Social Sciences, Brown University, School of Public Health, Providence, Rhode Island; 4 Behavioral Health Equity Research Group, Hennepin Healthcare Research Institute, Minneapolis, Minnesota; 5 Centers for Behavioral and Preventive Medicine, The Miriam Hospital, Providence, Rhode Island; 6 Department of Psychiatry and Human Behavior, Alpert School of Medicine, Brown University, Providence, Rhode Island; 7 Division of Clinical Pharmacology, Department of Medicine, Hennepin Healthcare, Minneapolis, Minnesota; 8 Department of Medicine, University of Minnesota, Minneapolis

## Abstract

**Importance:**

Tobacco use prevalence among individuals involved in the criminal-legal system is 125% higher than that of the general population and leads to high levels of smoking-related morbidity and mortality.

**Objective:**

To examine the acceptability, feasibility, and preliminary clinical outcomes of a smoking cessation intervention for individuals who are incarcerated.

**Design, Setting, and Participants:**

This pilot randomized clinical trial was conducted from January 2019 to May 2020. Participants were recruited in a pretrial county jail in a large Midwestern US city and were followed up after release. Participants were incarcerated, smoked daily before incarceration, desired to stay quit or reduce cigarette smoking upon release, and expected to be released to the community within 90 days of enrollment. Data analysis was performed from June to October 2020.

**Interventions:**

Participants randomized to the counseling plus nicotine replacement therapy (NRT) group received 1 hour of smoking cessation counseling in jail, a supply of nicotine lozenges upon release, and up to 4 telephone counseling sessions after release. Those randomized to brief health education (BHE) received 30 minutes of general health education in jail.

**Main Outcomes and Measures:**

The primary clinical outcome was biologically verified 7-day point prevalence abstinence (PPA) at 3 weeks after release. Secondary clinical outcomes included 7-day PPA at 12 weeks, changes in number of cigarettes per day, and time to smoking lapse and relapse.

**Results:**

A total of 46 participants (42 men [91%]; mean [SD] age, 38.2 [9.1] years) were enrolled and remained eligible at release; 23 were randomized to the counseling plus NRT group and 23 were randomized to the BHE group. Recruitment, enrollment, and retention of participants was feasible and acceptable. There were no significant differences in smoking abstinence between groups as determined by 7-day PPA at 3 weeks (adjusted 7-day PPA, 11.9% for counseling plus NRT vs 10.6% for BHE; odds ratio, 1.13; 95% CI, 0.14-9.07) and at 12 weeks (adjusted 7-day PPA, 11.1% for counseling plus NRT vs 14.3% for BHE; odds ratio, 0.75; 95% CI, 0.09-6.11). Cigarettes per day for the counseling plus NRT group decreased more compared with the BHE group at both 3 weeks (difference [SE], −4.58 [1.58] cigarettes per day; 95% CI, −7.67 to −1.48 cigarettes per day; *P* = .007) and 12 weeks (difference [SE], −3.26 [1.58] cigarettes per day; 95% CI, −5.20 to −0.20 cigarettes per day; *P* = .04) after release.

**Conclusions and Relevance:**

Initiation of counseling plus NRT during incarceration and continuing after release is feasible and acceptable to participants and may be associated with reduced cigarette use after release. However, additional supports are needed to increase engagement in telephone counseling after release. A larger clinical trial is warranted to determine the effectiveness of counseling plus NRT.

**Trial Registration:**

ClinicalTrials.gov Identifier: NCT03799315

## Key Points

**Question:**

What are the acceptability, feasibility, and preliminary clinical outcomes of counseling plus nicotine replacement therapy (NRT) in a pretrial, urban jail?

**Findings:**

In this randomized clinical trial, counseling plus NRT was acceptable and feasible. There was no difference in 7-day point prevalence abstinence at 3 weeks, but the reduction in cigarettes per day was significantly greater in the counseling plus NRT group compared with the control group.

**Meaning:**

These findings suggest that counseling plus NRT is feasible, and preliminary results indicate a potential reduction in smoking after release from jail; larger clinical trials are needed to determine the effectiveness of smoking cessation interventions in county jails.

## Teaser

This pilot randomized clinical trial examines the acceptability, feasibility, and preliminary clinical outcomes of a smoking cessation intervention for individuals who are incarcerated in a pretrial, urban jail.

## Introduction

Cigarette use in the US is concentrated among low-income populations and among individuals who are Black, Indigenous, or people of color.^1^ These populations are overrepresented in the US criminal-legal system, where smoking rates are 125% higher compared with the general population and contribute to excess morbidity and mortality.^2,3^ Although an increasing body of research has focused on opioid use among people involved in the criminal-legal system,^4,5^ little attention has been paid to tobacco use and cardiovascular disease, the second leading cause of death among recently incarcerated people.^6^ Reducing tobacco use among a population with elevated levels of premature mortality and that frequently uses high-cost health care services could be associated with substantial improvements in health and reductions in health care costs.^7^

Few clinical trials have examined smoking cessation treatment in correctional settings. One of the only tobacco-related trials^8^ conducted in the US targeted individuals incarcerated in a smoke-free prison for at least 6 months and randomized participants to a counseling only intervention or to treatment as usual (ie, forced abstinence). In that study,^8^ 93% of control and 75% of intervention participants relapsed by 3 weeks after release. Another trial^9^ in an all-female prison that allowed smoking found that women who received nicotine replacement therapy (NRT) and group counseling while incarcerated showed significantly greater rates of abstinence than a waitlist control group (14% vs 3% abstinence during incarceration). These results suggest that individuals who are incarcerated have interest in initiating smoking cessation, forced abstinence alone does little to influence postrelease smoking, and counseling and/or NRT started during incarceration can reduce rates of smoking.

To our knowledge, no trials have examined the impact of smoking cessation interventions in US county jails, which have much higher turnover than prisons. More than 10 million individuals leave local jails every year, compared with fewer than 1 million who leave prisons. Jail stays are generally short, and release is often unpredictable, which necessitates brief and flexible interventions.

We conducted a pilot randomized clinical trial (RCT) of counseling plus NRT initiated during incarceration and continued after release from jail vs brief health education (BHE). This pilot RCT was primarily designed to examine the feasibility and acceptability of conducting all aspects of the study protocol in a county jail. We also collected and report preliminary clinical outcome data.

## Methods

### Design and Setting

We followed Consolidated Standards of Reporting Trials (CONSORT) reporting guidelines for RCTs. All procedures for the Jail-Based Use of Smoking Treatment (JUST) study were approved by the Hennepin Healthcare Research Institute institutional review board. Written informed consent was obtained from all participants. The study protocol and statistical analysis plan are shown in Supplement 1.

This study was an RCT that enrolled participants at the Hennepin County Jail (hereafter referred to as jail), a tobacco-free pretrial jail in Minneapolis, Minnesota. An individual may stay at the jail from hours to a year (the median stay is 2 days). Some individuals leave without being charged, whereas others are charged and continue with court proceedings. Disposition upon release is often unpredictable (eg, release can be to community, treatment, or another correctional facility). Recruitment occurred for 30 weeks between January 2019 and February 2020, with study staff recruiting in the jail approximately 6 to 12 business hours per week. The final follow-up assessment was completed in May 2020.

### Participants

Participants were English-speaking individuals incarcerated in the jail who smoked cigarettes daily before admission. All participants were cleared for nicotine lozenge use by health care staff before referral to study staff. Participants were referred to the study team if they reported smoking before incarceration and expressed an interest in quitting smoking during screening by jail health care staff or if they self-referred after learning about the study. Participants were enrolled only if they expected to be released within 90 days of enrollment and to have regular telephone access after release. A detailed list of inclusion and exclusion criteria are reported in the eTable in Supplement 2.

### Randomization and Study Procedures

Participants were approached, screened, and consented and completed a baseline assessment and in-person treatment components in the jail health clinic. Screening, consent, assessment, and in-jail counseling and education were conducted in the jail by the same individual (ie, the counselors). In most cases, the baseline assessment and initiation of treatment occurred immediately after enrollment, although these occasionally took place 1 to 2 days after enrollment. Demographics, housing, substance use, physical and mental health status,^10^ cigarettes per day, and nicotine dependence^11^ were assessed at baseline. Race/ethnicity was self-reported by participants. Race/ethnicity was assessed in this study because of the high rates of tobacco use and criminal justice involvement among Black and Native American Minnesotans compared with White Minnesotans.

After the baseline assessment, participants were randomized to either counseling plus NRT or BHE. We used a permuted block randomization procedure with small, randomly sized blocks allocated in a 1:1 manner. Randomization was stratified by sex, and the scheme was generated in R statistical software version 3.6.1 (R Project for Statistical Computing).^12^ The study statistician provided a randomization sequence. This sequence was concealed from all other study staff and investigators, as well as participants. The counselor went to initial in-jail intervention visits prepared to initiate either condition. When the participant was in the room and ready to begin, the counselor randomized the participant via the randomization module in REDCap software version 10.0.33 (REDCap Project).

### Counseling Plus NRT

Participants in the counseling plus NRT group received 1 hour of in-person smoking cessation counseling during incarceration and up to 4 telephone sessions over the course of 3 weeks after release. Smoking cessation counseling sessions were guided by current clinical guidelines^13^ and were tailored to incorporate how smoking may interact with the stress of transitioning from jail to community.

During session 1, a counselor provided participants a rationale for the study, made a strong recommendation to make a quit attempt at release, assessed smoking goals, and formulated a written plan for a quit or reduction attempt at release (including strategies to mitigate triggers, manage cravings, and obtain social support). Participants sampled a 2-mg nicotine lozenge during session 1. Three packs of 2-mg nicotine lozenges (81 lozenges per pack) and instructions for use were put in participants’ property that was returned to them at their release.

Counseling plus NRT participants were called for 10- to 20-minute telephone sessions at 1, 7, 14, and 21 days after release by the same counselor who conducted counseling during incarceration. These sessions included monitoring of smoking status and lozenge use, goal setting and problem-solving regarding the plan to quit or reduce smoking, and emotional support and brief problem-solving regarding stress after release from jail.

### Brief Health Education

Participants randomized to BHE received 30 minutes of general health education in person during incarceration. The content was focused on exercise, diet, tobacco use, and sexual health. BHE participants who were still smoking at the 12-week assessment were offered a free supply of nicotine lozenges.

### Counselor Training and Treatment Fidelity

Each study condition had a detailed manual. Two counselors provided care in both conditions. One was a certified community health worker (not one of the coauthors of this article) and the other was a bachelor’s-degree level social worker (E.S.). Counselors completed a comprehensive training program on how to implement each manual, including a Tobacco Treatment Specialist course accredited by the Council for Tobacco Treatment Training Programs.

One of the authors (T.N.A.W.) provided supervision of the BHE condition, and another author (A.M.B.) provided supervision for the counseling plus NRT condition. For all sessions in both conditions, the counselor completed a treatment fidelity checklist. Counseling sessions in both conditions were audio recorded, and a random 20% of sessions were coded by supervisors using the treatment fidelity checklist.

### Outcomes

#### Feasibility and Acceptability

Study feasibility was assessed using recruitment rate per week, receipt of education or counseling, receipt and use of study-provided NRT, and percentage of follow-up assessments completed. Intervention acceptability was measured at week 12 by the Client Satisfaction Questionnaire–8,^14^ which has a range of 8 to 32, with higher scores indicating greater acceptability. We conducted brief, semistructured interviews with 8 participants across both conditions for qualitative feedback.

#### Smoking Outcomes

Follow-up assessments were completed in person or by telephone at 1, 3, and 12 weeks after release by an assessor who was blind to condition and did not have previous contact with the participant. The primary clinical outcome of interest was biologically verified 7-day point prevalence abstinence (PPA), which was defined as no smoking, not even a puff, in the previous 7 days and was confirmed using a carbon monoxide monitor (<8 ppm was considered abstinence) at 3 weeks after release. We used a carbon monoxide detector (piCO^+^; coVita) that was calibrated according to manufacturer instructions. Secondary outcomes included biologically verified 7-day PPA at 12 weeks, change in self-reported cigarettes per day at 3 and 12 weeks, time to first cigarette after release, and time to relapse after release. Relapse was defined as smoking on 7 consecutive days or in 2 consecutive 7-day periods.

### Statistical Analysis

A priori, we chose to withdraw participants who remained incarcerated longer than 90 days after enrollment and to exclude these participants from analysis as ineligible after randomization. We planned to enroll 60 participants (with the expectation that 50 would remain eligible after randomization) to have sufficient feasibility and acceptability data to support a future fully powered trial.

Baseline characteristics of participants in each condition were compared using 2-sided *t* tests, χ^2^ tests, and other nonparametric tests (for skewed and low cell count variables) as appropriate. Variables that differed significantly between groups and were associated (at a *P* < .10 level) with the outcome of interest were included in multivariable models (generalized estimating equations, generalized linear models, and Cox models as appropriate). Finally, we examined the sensitivity of effect estimates to the inclusion of these baseline variables in our final models. Point estimates did not change with the inclusion of confounders. We present adjusted results (ie, adjusted for marijuana use before incarceration) in all smoking outcome models.

A longitudinal model implemented with generalized estimating equations with robust SEs was used to estimate treatment effects on 7-day PPA at 3 and 12 weeks after release. Models used a logit link and Bernoulli distribution and considered odds ratios (ORs) and 95% CIs as measures of effect size. Next, a generalized linear model with identity link was used to estimate treatment associations with cigarettes per day at 3 and 12 weeks after release. This linear model was adjusted for baseline smoking rate. Finally, a Cox proportional hazards model was used to estimate associations of treatment with time to first cigarette and time to relapse. Hazard ratios (HRs) and 95% CIs were considered measures of effect size in this case.

All models were run on the intent-to-treat sample with all participants released in 90 or fewer days included in the analysis. Likelihood-based and quasi-likelihood–based approaches to estimation were used, which make full use of available data without directly imputing missing values to provide stable estimates of the regression parameters. Data analysis was performed with SAS statistical software version 9.3 (SAS Institute). Data analysis was performed from June to October 2020.

## Results

### Sample and Baseline Characteristics

**Figure 1.**
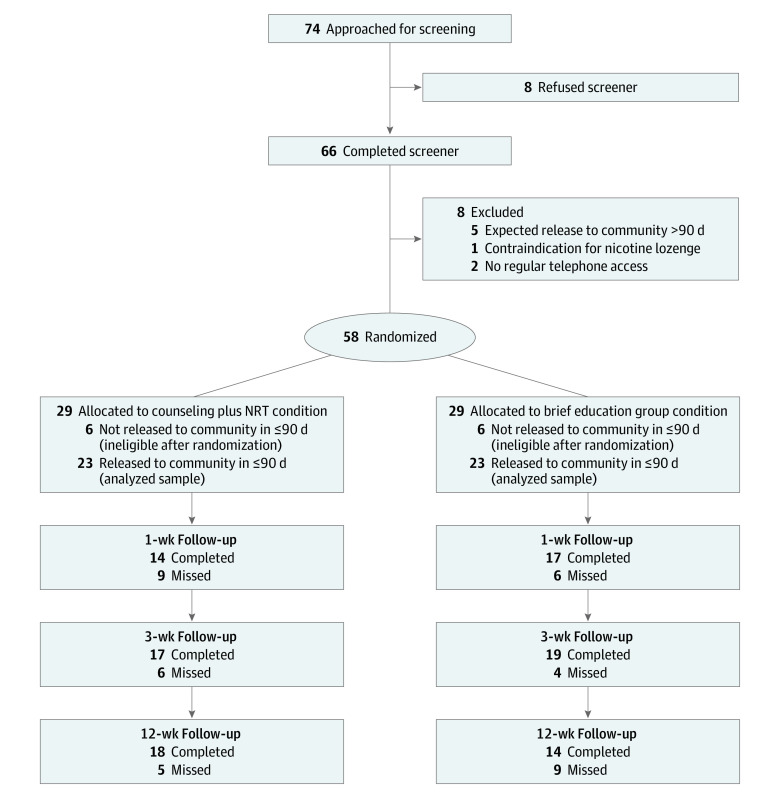
CONSORT Diagram of Participant Flow Through Trial NRT indicates nicotine replacement therapy.

A total of 74 participants were approached in person, 66 completed screening, and 58 were randomized. Recruitment was ended after 58 participants because in-jail research was suspended as a result of the COVID-19 pandemic. In each condition, 29 participants were randomized, 23 of whom remained eligible after randomization and were included in analyses. The consort diagram in Figure 1 provides details of exclusions during the study and the final sample. All results reported here are for the analyzed sample of 46 participants.

Sample characteristics are provided in the Table. In general, groups were well balanced. Our sample was predominantly male (42 men [91%]) and unstably housed (30 participants [65%] did not rent or own their own homes). Only 28% of the sample (13 participants) identified as White. The mean (SD) age of participants was 38.2 (9.1) years. More than one-half of participants (26 participants [57%]) reported methamphetamine use in the week before jail admission and more than one-quarter (13 participants [28%]) reported heroin use. Substantially more participants in the counseling plus NRT group than in the BHE group used marijuana in the week before jail admission (14 participants [61%] vs 11 participants [48%]).

**Table.** Baseline Characteristics of Cohort

Characteristic	Participants, No. (%)	
	Counseling plus NRT (n = 23)	BHE (n = 23)
Age, mean (SD), y	36.3 (8.8)	40.0 (9.2)
Sex		
Male	21 (91)	21 (91)
Female	2 (9)	2 (9)
Hispanic or Latino ethnicity	4 (17)	3 (13)
Race^a^		
White	7 (30)	6 (26)
Black or African American	8 (35)	5 (22)
American Indian or Alaskan Native	2 (9)	5 (22)
Asian	1 (4)	0
Pacific Islander	1 (4)	0
Other^b^	4 (17)	7 (30)
Housing situation before jail		
Homeowner or renter	9 (39)	7 (30)
Living with friends, relatives, or partner	8 (35)	6 (26)
Shelter or no steady place to stay	6 (26)	10 (44)
Drug use in week before jail		
Marijuana^c^	14 (61)	11 (48)
Cocaine	5 (22)	5 (22)
Heroin	6 (26)	7 (30)
Methamphetamine	13 (57)	13 (57)
Prescription pain relievers	4 (17)	3 (13)
Fagerstrom Test for Nicotine Dependence^11^ score, mean (SD)	5.87 (2.03)	5.30 (1.55)
12-Item Short-Form Health Survey^10^ score, mean (SD)		
Mental health summary score	43.62 (9.89)	44.36 (11.14)
Physical health summary score	50.42 (6.78)	48.42 (8.10)

Abbreviations: BHE, brief health education; NRT, nicotine replacement therapy.

^a^ Race was self-reported by participants.

^b^ Other refers to any other racial category as self-reported by the participants, including multiple races.

^c^ Significant difference between groups at *P* = .04.

### Feasibility and Acceptability

Individuals approached for screening were generally eager to participate, and 1.9 participants were enrolled per week. All participants received in-jail counseling or education as planned. Among counseling plus NRT participants released to the community, all received study-provided NRT, and 14 (60.8%) completed at least 1 telephone counseling session (mean [SD] telephone sessions completed, 1.2 [1.2] sessions). All 14 participants who completed a telephone counseling call reported using study-provided lozenges.

Forty-one participants (89.1%) completed at least 1 assessment after release (completion rates, 67.4% at 1 week, 78.3% at 3 weeks, and 69.6% at 12 weeks). Reasons for missed counseling and assessments varied, but homelessness (5 participants reported homelessness during the study), reincarceration (13 participants missed assessments because of reincarceration), unreliable telephone access (15 participants provided telephone numbers that did not work or changed frequently), and difficulty contacting participants in drug rehabilitation settings (8 participants missed assessments while in residential drug treatment) were common barriers. The mean (SD) Client Satisfaction Questionnaire–8 score was 28.1 (2.5) in the counseling plus NRT group, indicating high treatment acceptability.

Eight participants completed exit interviews with the study coordinator (B.R.F.). Participants from both conditions expressed a positive impression of the in-jail counseling or education. When asked about use of lozenges, participants in the counseling plus NRT group expressed that it would have been helpful to receive lozenges regularly while in jail, especially immediately before release to control cravings during the stressful transition. Participants who were released from jail to a drug treatment facility described barriers to quitting that may be unique to treatment settings, including social pressure to smoke with other residents.

Participants also provided insights on barriers to engaging in assessments or counseling. In particular, telephone access to complete calls was challenging to participants in the community (eg, limited minutes, shared telephone with family members) and residential drug treatment (eg, limited free time or treatment program limits on daily telephone time).

### Treatment Fidelity

In the BHE condition, counselors documented completing 100% of planned components. Supervisor-rated BHE sessions also indicated 100% fidelity. In the counseling plus NRT condition, counselors documented completing 91.7% of planned components. Supervisor ratings of fidelity in the counseling plus NRT condition indicated 92.6% fidelity.

### Smoking Outcomes

#### 7-Day PPA

At 3 weeks, 7-day PPA did not differ significantly between groups (OR, 1.13; 95% CI, 0.14-9.07; adjusted 7-day PPA, 11.9% for counseling plus NRT vs 10.6% for BHE). Similarly, model results did not indicate a significant between-group difference in 7-day PPA at 12 weeks (OR, 0.75; 95% CI, 0.09-6.11; adjusted 7-day PPA, 11.1% for counseling plus NRT vs 14.3% for BHE). Models controlled for marijuana use, which was significantly associated with 7-day PPA (χ^2^_1_ = 2.17; *P* = .04) and significantly different between groups (χ^2^_1_ = 2.90; *P* = .04) (Table).

#### Smoking Rate

**Figure 2.**
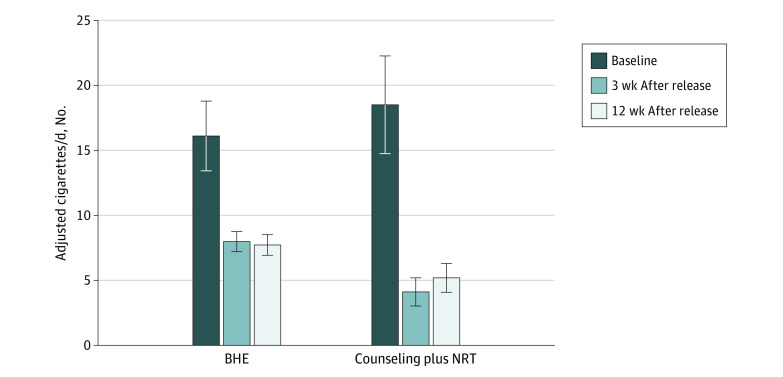
Cigarettes per Day Over Time BHE indicates brief health education; NRT, nicotine replacement therapy.

Figure 2 shows the number of cigarettes used per day before and after incarceration in both groups. There was a significant difference between groups in change in cigarettes per day from baseline to 3 weeks, with counseling plus NRT participants reducing their cigarettes per day significantly more than BHE participants, adjusting for marijuana use before jail (difference [SE], −4.58 [1.58] cigarettes per day; 95% CI, −7.67 to −1.48 cigarettes per day; *P* = .007). A similar pattern of findings was seen at 12 weeks, with counseling plus NRT participants reporting greater reductions in cigarettes per day compared with BHE participants (difference [SE], −3.26 [1.58] cigarettes per day; 95% CI, −5.20 to −0.20 cigarettes per day; *P* = .04).

#### Time to First Cigarette

There were no significant differences between groups with respect to time to first cigarette. The median (range) time to first cigarette was 1 (1-14) days for counseling plus NRT participants vs 1 (1-2) days for BHE participants. At 3 weeks, the HR was 0.58 (95% CI, 0.12-2.89), which indicates a lower instantaneous risk of smoking for counseling plus NRT vs BHE participants (although the difference was not significant). A similar HR was observed at 12 weeks (0.67; 95% CI, 0.14-2.98).

#### Time to Relapse

Models did not suggest significant between-group differences in time to relapse. The median (range) time to relapse was 8 (8-22) days for the counseling plus NRT participants vs 7 (7-8) days for BHE participants. HRs for time to relapse were 0.83 (95% CI, 0.39-1.74) at 3 weeks and 0.82 (95% CI, 0.41-1.64) at 12 weeks.

## Discussion

In this pilot RCT of a smoking cessation intervention in an urban, pretrial county jail, we found that recruitment, enrollment, and retention of participants was feasible and acceptable. Our recruitment rate of approximately 2 participants per week, in addition to self-reported satisfaction with study participation, indicates that individuals who are incarcerated are interested in and enthusiastic about participating and that our procedures are feasible in a jail setting. We also found that 60.8% of counseling plus NRT participants completed at least 1 counseling session after release and 89.1% of participants completed at least 1 assessment. Taken together, these data suggest that conducting rigorous smoking cessation research initiated in pretrial correctional facilities and continued after release is feasible and acceptable to participants.

In preliminary outcome results, counseling plus NRT did not significantly increase abstinence from smoking over the 3 weeks following release from jail. However, we found significantly greater reductions in cigarettes per day among counseling plus NRT participants than among BHE participants at both 3 and 12 weeks after release. The extent of harm reduction achieved by smoking reduction is an ongoing debate. However, the magnitude of the reduction observed in this study indicates that important harm reduction may have been achieved. Time to first cigarette and time to relapse analyses did not show significant between-group differences. This preliminary evidence indicates that collection of outcome data is feasible following release from our county jail and that our brief intervention may have important effects on smoking reduction upon release from jail.

There were several key findings from this pilot study that can inform future efforts to reduce tobacco use and associated morbidity among individuals involved in the criminal-legal system. First, reincarceration, residential drug treatment, and poor telephone access affected postrelease follow-up. These issues could be addressed in future work by re-engaging participants during periods of reincarceration, establishing formal relationships with treatment centers, and providing telephone access through the study when needed. Second, most participants reported illicit substance use before their jail admission, highlighting the importance of incorporating smoking cessation treatment with other substance use treatment. Finally, participants expressed the importance of developing the habit of using nicotine lozenges before cigarettes were available to them, especially access to lozenges in the hours before release. This finding suggests that providing NRT throughout an incarceration stay, including immediately before release, could be associated with prevention of the high rates of returning to smoking within hours of release. This feedback from participants is consistent with previous work on the importance of having the opportunity to sample NRT before a quit attempt^15^ and work showing that short-acting NRT is more effective if used in anticipation of situations that will trigger cravings (eg, release from incarceration).^16^

### Limitations

Our study has several limitations. First, our primary outcome was feasibility and acceptability, and all clinical outcomes were underpowered. Thus, all clinical outcomes should be treated as preliminary, and conclusive results await a fully powered clinical trial. Second, we conducted exit interviews during assessments and, thus, participants who missed assessments were not interviewed. Third, we learned through informal interactions that some drug treatment facilities would not allow participants to use study-provided NRT while in residential treatment. We plan to mitigate this problem in future studies by working with treatment centers to allow NRT use.

## Conclusions

Tailored smoking cessation programs designed for individuals leaving incarceration are needed to reduce disparities in smoking and associated morbidity and mortality. The results of this study show that providing counseling plus NRT to individuals while they are in jail is feasible and acceptable to participants and may be associated with reduced cigarette use after release. A larger clinical trial is warranted to determine the effectiveness of counseling plus NRT during the transition from jail to the community.

## Notes

Supplement 1. Trial Protocol and Statistical Analysis Plan

Supplement 2. **eTable.** Inclusion and Exclusion Criteria

Supplement 3. Data Sharing Statement

## References

[1] Schroeder SA. American health improvement depends upon addressing class disparities. Prev Med. 2016;92:6-15. doi:10.1016/j.ypmed.2016.02.024 27018943

[2] Winkelman TNA, Vickery KD, Busch AM. Tobacco use among non-elderly adults with and without criminal justice involvement in the past year: United States, 2008-2016. Addict Sci Clin Pract. 2019;14(1):2. doi:10.1186/s13722-019-0131-y 30635028 PMC6329085

[3] Puljević C, Segan CJ. Systematic review of factors influencing smoking following release from smoke-free prisons. Nicotine Tob Res. 2019;21(8):1011-1020. doi:10.1093/ntr/nty088 29733380

[4] Green TC, Clarke J, Brinkley-Rubinstein L, . Postincarceration fatal overdoses after implementing medications for addiction treatment in a statewide correctional system. JAMA Psychiatry. 2018;75(4):405-407. doi:10.1001/jamapsychiatry.2017.4614 29450443 PMC5875331

[5] Winkelman TNA, Chang VW, Binswanger IA. Health, polysubstance use, and criminal justice involvement among adults with varying levels of opioid use. JAMA Netw Open. 2018;1(3):e180558. doi:10.1001/jamanetworkopen.2018.0558 30646016 PMC6324297

[6] Binswanger IA, Blatchford PJ, Mueller SR, Stern MF. Mortality after prison release: opioid overdose and other causes of death, risk factors, and time trends from 1999 to 2009. Ann Intern Med. 2013;159(9):592-600. doi:10.7326/0003-4819-159-9-201311050-00005 24189594 PMC5242316

[7] de Andrade D, Kinner SA. Systematic review of health and behavioural outcomes of smoking cessation interventions in prisons. Tob Control. 2016;26(5):495-501. doi:10.1136/tobaccocontrol-2016-053297 27798322 PMC5574402

[8] Clarke JG, Stein LA, Martin RA, . Forced smoking abstinence: not enough for smoking cessation. JAMA Intern Med. 2013;173(9):789-794. doi:10.1001/jamainternmed.2013.197 23567902 PMC4438989

[9] Cropsey K, Eldridge G, Weaver M, Villalobos G, Stitzer M, Best A. Smoking cessation intervention for female prisoners: addressing an urgent public health need. Am J Public Health. 2008;98(10):1894-1901. doi:10.2105/AJPH.2007.128207 18703440 PMC2636452

[10] Ware J Jr, Kosinski M, Keller SDA. A 12-Item Short-Form Health Survey: construction of scales and preliminary tests of reliability and validity. Med Care. 1996;34(3):220-233. doi:10.1097/00005650-199603000-00003 8628042

[11] Heatherton TF, Kozlowski LT, Frecker RC, Fagerström KO. The Fagerström Test for Nicotine Dependence: a revision of the Fagerström Tolerance Questionnaire. Br J Addict. 1991;86(9):1119-1127. doi:10.1111/j.1360-0443.1991.tb01879.x 1932883

[12] R Core Team. R: a language and environment for statistical computing. R Foundation for Statistical Computing. Published 2013. Accessed May 25, 2021. http://www.R-project.org/

[13] Fiore MC, Jaen CR, Baker TB, . Treating tobacco use and dependence: 2008 update. US Department of Health and Human Services. Published 2008. Accessed April 12, 2021. https://www.ncbi.nlm.nih.gov/books/NBK63952/

[14] Nguyen TD, Attkisson CC, Stegner BL. Assessment of patient satisfaction: development and refinement of a service evaluation questionnaire. Eval Program Plann. 1983;6(3-4):299-313. doi:10.1016/0149-7189(83)90010-1 10267258

[15] Burris JL, Heckman BW, Mathew AR, Carpenter MJ. A mechanistic test of nicotine replacement therapy sampling for smoking cessation induction. Psychol Addict Behav. 2015;29(2):392-399. doi:10.1037/adb0000035 25347021 PMC4411194

[16] Kotlyar M, Lindgren BR, Vuchetich JP, . Timing of nicotine lozenge administration to minimize trigger induced craving and withdrawal symptoms. Addict Behav. 2017;71:18-24. doi:10.1016/j.addbeh.2017.02.018 28235705 PMC5449230

