# Supplementary material for: Feasibility and Acceptability of a Smoking Cessation Program for Individuals Released From an Urban, Pretrial Jail: A Pilot Randomized Clinical Trial
Source: JAMA Netw Open. 2021 Jul 6;4(7):e2115687. doi: 10.1001/jamanetworkopen.2021.15687 (PMC8261607; doi:10.1001/jamanetworkopen.2021.15687)
Supplement: Supplement 1. — Trial Protocol and Statistical Analysis Plan [file jamanetwopen-e2115687-s001.pdf]

Reducing Tobacco-Related Health Disparities Among Incarcerated Individuals in Hennepin County

PROTOCOL

Purpose:

1) Does counselling plus nicotine replacement therapy (C+NRT) improve bio-verified smoking cessation rates over three weeks post-discharge compared to brief health education (BHE)?

2) Does C+NRT increase time-to-lapse and time-to-relapse relative to BHE?

3) Does C+NRT improve smoking cessation rates over 12 weeks compared to BHE?

4) Does C+NRT improve health-related quality of life (HRQoL), mental health, and substance use?

Process for Information Exchange:

Dr. Winkelman and research staff will meet with the Adult Detention Center (ADC) Medical Team, including the Medical Director, the nurse manager, and other key medical staff, every other Tuesday during the Medical Team standing meeting to provide updates and discuss any challenges regarding study recruitment and implementation. Regular communication by phone and email between these meetings will allow for timely communication of any unanticipated problems. Dr. Winkelman will also have regular weekly visits at the Hennepin County (ADC) where study participants are screened and recruited for study eligibility by research staff.

Privacy Guidelines:

Research staff and health care providers involved in the study will have access to participants' electronic medical records. Because the medical record will be reviewed as part of the recruitment process and to review an individual's current incarceration status as part of follow-up scheduling, we will apply for a Protected Health Information (PHI) waiver from the MMRF IRB. All data will be treated as confidential and will never be stored or reported in association with identifying information. All study data will be entered directly into Research Electronic Data Capture system (REDCap) software and securely encrypted. Only the PI, co-PI, and study staff who have completed MMRF IRB approved Human Subjects Research and Good Clinical Practice training through the Collaborative Institutional Training Initiative (CITI) will have access to identifiable information. All data will be de-identified and filed according to a participant study ID on an encrypted private server that can only be accessed by the PI, co-PI, and research staff. Any forms with identifying information will be stored in a locked cabinet in a locked office within a secure research unit with limited access to small number of MMRF researchers. Only de-identified data will be published.

We have received a Certificate of Confidentiality through the United States Department of Health and Human Services to ensure that collected data cannot be used in legal proceedings.

Informed Consent:

The study will be verbally described in detail by study staff to each subject using the approved informed consent documents, providing ample opportunity for participants to ask questions relating to their involvement in the study. Subjects will be self-consenting to participate in the study. Patients will be screened by research staff for pre-incarceration smoking habits while they are waiting for their routine health assessment visit in a holding cell next to the clinic examination rooms. In order to minimize the possibility of coercion or the undue influence this may bring, the study will be described in full, including the follow-up requirements, and time commitment involved in participation. This will allow potential subjects to weigh the potential benefit versus the costs of their time and effort. Before consenting, participants will be given a simple true/false test to ensure that they understand the study procedures. After consent, subjects will be asked to fill out the contact information form and a release of information form (to be used in the case that they are released to another facility, such as the Adult Corrections Facility or a residential treatment facility). The study will be administered to English speaking subjects only.

Inclusion Criteria:

1) use of ≥ 1 cigarette per day prior to incarceration, 2) age 18-64, 3) English fluency, 4) lives within 20 minutes of Hennepin County Medical Center and has no plans to move away from area for 4 months, 5) willing to attempt quitting or reducing smoking at discharge, 6) has a telephone, 7) expected release from the Hennepin Adult Detention Center to the community within 90 days, 8) cleared for nicotine lozenge safety by jail health care provider and willing to use at discharge.

Exclusion Criteria:

1) active tuberculosis, 2) current mental health crisis (i.e., currently experiencing significant mania, psychosis, or suicidality), 3) unable to ambulate independently, 4) acute medical condition that would impair their ability to follow-up for assessments, 5) expected discharge to a controlled institutional setting (e.g., locked state mental health facility, inpatient treatment facility, prison), 6) active pregnancy, 7) myocardial infarction within last 2 weeks, 8) indication in medical chart that the patient may be a threat to study staff, 9) been incarcerated for more than 30 days.

Recruitment:

We intend to recruit a sample of 50 total patients, and randomize 25 into the experiment and 25 to the control group. Patients will be screened by research staff for pre-incarceration smoking habits while they are waiting for their routine health assessment visit in a holding cell next to the clinic examination rooms. Due to the pre-sentence nature of jail, it is likely that a number of subjects will be ineligible post-randomization due to the length of their sentence. Participants who have not yet been released to the community 12 weeks after enrollment will be removed from the JUST study. As a result, we intend to continue recruitment until we have 50 patients whose sentences meet the criteria of the study, acknowledging that our gross sample size may be larger than 50. The Hennepin County ADC clinic is located on the fourth floor of the ADC and is staffed by medical professionals, including physicians, nurse practitioners, and registered nurses.

Guards are present to ensure safety of staff and patients. We expect at least 40% of the 4500 unique patients seen in clinic per year will be current, daily smokers.

Enrollment:

Research staff will discuss inclusion and non-health related exclusion criteria with participants. Eligible patients will be consented for inclusion into the study. Health-related exclusion criteria will be verified by a registered nurse following a health assessment. Patients will be cleared to use the nicotine lozenge by a registered nurse, following a health assessment, if the patient meets all inclusion criteria and meets no exclusion criteria. In the case that the health assessment is conducted by a nurse who is not listed as study personnel, a physician who is listed as study personnel will review the patient's chart to determine eligibility. The physician will either sign the screener form or give verbal approval and have study staff sign the form on their behalf. Indeterminate cases will be discussed on an individual basis with an on-call physician (Dr. Winkelman) immediately following the health assessment. Only patients for whom the potential benefit of smoking cessation outweighs any medical risk will be enrolled, as determined by a registered nurse or physician. Following enrollment, individuals will be scheduled for a counseling session within 72 hours, possibly the same day. Those randomized to the treatment group will be offered the chance to sample a nicotine lozenge.

Measured Variables:

Participants will complete self-reported baseline and follow-up assessments including the following items:
- smoking in the past week (self-report and Fagerstrom Test for Nicotine Dependence)
- general health (SF-12)
- physical health (SF-12)
- mental health (SF-12, CESD-10, PANAS)
- substance use (DAST-10, marijuana, cocaine, heroin, methamphetamine, prescription pain relievers, or binge drinking [4/5 drinks on the same occasion] in past 7 days)
- physical activity and diet
- hospitalizations, emergency department visits, and jail stays
- blood pressure and BMI
- nicotine lozenge use

Participants who deny cigarette use in the past 7 days will undergo carbon monoxide breath testing during follow-up assessments.

Safety Provisions:

Pending IRB review, our study is likely to meet the definition of "minimal risk" for research involving prisoners: "Minimal risk is the probability and magnitude of physical or psychological harm that is normally encountered in the daily lives, or in the routine medical, dental, or psychological examination of healthy persons." (45 CFR 46.303(d))The Office for Human Research Protections interprets "healthy persons" to mean healthy persons who are not prisoners.

However, measures are in place to ensure the safety of all participants, as well as the validity and integrity of the data.

Patient safety monitoring begins with the health-related exclusion criteria, which requires a registered nurse who has access to the participant's electronic medical records to review and clear the patient for use of nicotine lozenges following a health assessment. Indeterminate cases will be discussed on an individual basis with an on-call physician (Dr. Winkelman) immediately following the health assessment. Only patients for whom the potential benefit of smoking cessation outweighs any medical risk will be enrolled, as determined by a registered nurse or physician.

Additionally, the Co-PI will monitor recordings of intervention counseling sessions, and staff will be trained in responding to extreme depressive symptoms, suicidality, and medical emergencies. Monitoring of adverse events will be continuous and will be conducted under the direction of the Principal Investigator.

Patients who report suicidal ideation will be referred to the Acute Psychiatric Services clinic.

Remuneration:

All participants will receive $40 cash for each assessment completed, and an additional $30 cash bonus for completion of all three assessments. If necessary, a subject may complete an assessment over the phone. If the assessment is completed over the phone, the participant may only pick up his/her payment in person within 7 days (2 days preferred) of the assessment if they reported not smoking; if the participant reported smoking, the payment may be sent to the participant as a check or in cash, or the participant may pick up his/her payment from the Berman center by the end of their participation window. Assessment procedures will be identical for participants in both the experimental and control groups. However, following completion of the 12 week assessment, those in the control group will be offered nicotine lozenges if they are still smoking, at no cost to them. Participants will either arrange their own transportation to the Berman Center or other in-person assessment locations, including Hennepin Healthcare clinical care sites, or have study staff order a taxi for them (if staff find that they qualify for this latter option). Participants who arrange their own transportation will be paid an additional $5 to reimburse their travel costs. In addition, some participants will be offered $20 for completing an exit interview with study staff.

Summary:

Smoking rates remain above 60% for individuals involved in the criminal justice system and contribute to excess morbidity and mortality. Addressing smoking disparities among justice-involved individuals is a critical public health issue in Minnesota. Minnesota is one of only a few states with rising incarceration rates. People who are incarcerated represent the intersection of multiple high-priority populations (disproportionately African-American, Native American, low-income, homeless, on Medicaid, and suffering from mental illness and substance use disorders). We propose to examine the impact of an intervention on bio-verified seven-day point prevalence abstinence for justice-involved individuals following discharge from jail. Participants will be

randomized to either guideline-based smoking cessation counseling during and after incarceration plus nicotine replacement or enhanced treatment as usual. Outcome assessments will be conducted for both arms at 1 week, 3 weeks, and 12 weeks post discharge from jail. They will occur over the phone or in-person at the Berman Center, in other Hennepin Healthcare clinical care sites (including jail health services), or at other community locations, such as treatment centers, shelters, or drop-in centers. Seven-day point prevalence abstinence will be bio-verified with exhaled carbon monoxide. Our analysis is powered (i.e., power > .8) to detect significant between group effects on our primary outcome (i.e., the longitudinal, between group effect on bio-verified seven-day point prevalence abstinence over the 3 weeks post-discharge). All analyses will be conducted on the intent to treat sample, with all randomized participants included in the analysis, and will utilize pre-specified logistic and linear regression models. In addition, some participants will be asked to participate in a phone or in-person exit interview with study staff after their 3 week or 12 week assessment. The interview will be used to help the study team improve their study designs in the future. The topics will include best modalities to use to communicate with participants, participants' impressions of the treatment content, and ideas participants have for other interventions that would benefit justice-involved individuals. We will use our findings to develop a larger, multi-site study that is fully powered to measure longer-term health and smoking cessation outcomes. If this larger trial demonstrates efficacy, we will lead an effort to implement this program in jails across MN.

Table.1 C+NRT and BHE protocol summaries

C+NRT	BHE
**During Incarceration**	
1-hour smoking cessation counseling (in-jail)	30 minutes of general health counseling (in-jail)
**Discharge from the Adult Detention Center**	
Nicotine lozenges provided upon discharge	No nicotine replacement therapy provided upon discharge
**Post-Incarceration**	
Phone counseling sessions<br>Day 1<br>Day 7<br>Day 14<br>Day 20	Phone counseling sessions<br>None
Assessments<br>Week 1* ($40 paid+$5 for transportation OR JUST-provided taxi)<br>Week 3* ($40 paid+$5 for transportation OR JUST-provided taxi)<br>Week 12* ($40 paid+$30 bonus+$5 for transportation OR JUST-provided taxi)<br>Possible exit interview with study personnel ($20)	Assessments<br>Week 1* ($40 paid+$5 for transportation OR JUST-provided taxi)<br>Week 3* ($40 paid+$5 for transportation OR JUST-provided taxi)<br>Week 12* ($40 paid+$30 bonus+$5 for transportation OR JUST-provided taxi)<br>Possible exit interview with study personnel ($20)

After Study Completion	
No additional procedures	Nicotine replacement provided for smokers at end of study

*The acceptable window for the week 1 assessment is +/-7 days, but +/-2 days is greatly preferred and should be encouraged. The acceptable window for the week 3 assessment is +5 weeks/-1 week, but +/- 7 days should be encouraged. The acceptable window for the 12 week assessment is +8 weeks/-4 weeks, but +/- 7 days should be encouraged.

Justification:

To our knowledge, only one US trial ("Project WISE") has evaluated the impact of a smoking cessation intervention provided during incarceration. This trial required a prison incarceration of at least 6 months. Results from this counseling-only intervention (i.e., no medication provided) indicated 93% of control participants and 75% of intervention participants relapsed by 3 weeks post discharge. By three months post discharge, 97% of control participants and 88% of intervention participants relapsed. These results suggest 1) forced abstinence during incarceration alone does little to influence post-discharge smoking, 2) counseling started during incarceration and continued after discharge can reduce smoking, and 3) more intensive treatment (e.g., counseling plus medication) may be needed to improve intervention effectiveness. A senior investigator on the WISE study (Dr. Bock) is a consultant on this proposal.

No trials have examined the impact of smoking cessation interventions in a county jail setting. Jails are a higher impact setting because more than 10 million individuals spend time in county jails every year, compared to about 2 million in prisons. However, jail stays are generally shorter (days to months) and discharge is often unpredictable, which necessitates interventions that are brief and flexible.

We propose to randomize smokers in the Hennepin County jail (Adult Detention Center–ADC) to guideline-based smoking cessation counseling plus nicotine replacement therapy (C+NRT) vs. brief health education (BHE). Findings from this study will support an application for a larger scale trial that utilizes an attention-matched control group and assesses the effect of smoking cessation on downstream health and health care utilization outcomes. The ultimate goal would be an effective treatment that could be disseminated across Minnesota correctional facilities.

Statistical Analysis Plan

Primary Analysis

All analyses will be conducted on the intent to treat sample, with all randomized participants included in the analysis. We will estimate the effect of the intervention on smoking cessation (biochemically verified 7-day point prevalence abstinence) over the 3 weeks post-release using a single repeated measures regression model implemented with generalized

estimating equations (GEE) and robust standard errors. Specifically, we will regress smoking status on intervention group (C+NRT vs. BHE) and potential covariates using binomial errors, a logit link function, and a working unstructured correlation to accommodate within-subject correlation. We will use similar methods to measure the effect of C+NRT at three months, although our studied is not powered to determine an effect at this time point.

We will examine time to event outcomes using Cox proportional hazards models. Finally, self-reported measures will be evaluated using descriptive statistics, as well as bivariate and multivariable logistic (for dichotomous outcomes) and linear regression models (for continuous outcomes).
