# Supplementary material for: Feasibility and Acceptability of a Smoking Cessation Program for Individuals Released From an Urban, Pretrial Jail: A Pilot Randomized Clinical Trial
Source: JAMA Netw Open. 2021 Jul 6;4(7):e2115687. doi: 10.1001/jamanetworkopen.2021.15687 (PMC8261607; doi:10.1001/jamanetworkopen.2021.15687)
Supplement: Supplement 2. — eTable. Inclusion and Exclusion Criteria [file jamanetwopen-e2115687-s002.pdf]

Supplemental Online Content

Winkelman TNA, Ford BR, Dunsiger S, et al. Feasibility and acceptability of a smoking cessation program for individuals released from an urban, pretrial jail: a pilot randomized clinical trial. *JAMA Netw Open*. 2021;4(7):e2115687. doi:10.1001/jamanetworkopen.2021.15687

eTable. Inclusion and Exclusion Criteria

This supplemental material has been provided by the authors to give readers additional information about their work.

© 2021 Winkelman TNA et al. *JAMA Network Open.*

eTable. Inclusion and Exclusion Criteria

**Inclusion Criteria**	
1	Incarcerated at the Hennepin County Jail for fewer than 30 days at time of consent
2	Used of ≥ 1 cigarette per day prior to incarceration
3	Age 18-64
4	English fluency
5	Live within 20 minutes of transit from the site of follow-up assessments (Hennepin County Medical Center) and no plans to move away from the area for 4 months
6	Willing to make an attempt to quit smoking at release or reduce smoking at release as a step towards future cessation
7	Expected access to a telephone upon release
8	Release from incarceration within 90 days of enrollment*
9	Cleared for nicotine lozenge safety by self-report and jail health care provider review (e.g., not pregnant)
10	Willing to use nicotine lozenges at release
**Exclusion Criteria**	
1	Mental or physical health condition that would have made participation unsafe or infeasible (i.e., active tuberculosis, significant mania, psychosis, or suicidality, unable to ambulate independently, other acute medical condition that would impair participation)
2	Expected release or transfer to a controlled institutional setting that would prevent participation after leaving jail (e.g., released to a locked state mental health facility or inpatient treatment facility, transfer to other jail or prison setting)
3	Indication in medical chart that the patient may be a threat to study staff

*Note that *expected release* timeline was used as a rule out on the screener. *Actual release* date of those enrolled was used to determine exclusion from analysis as ineligible post-randomization

© 2021 Winkelman TNA et al. *JAMA Network Open.*
