# Supplementary material for: Feasibility and Acceptability of a Smoking Cessation Program for Individuals Released From an Urban, Pretrial Jail: A Pilot Randomized Clinical Trial
Source: JAMA Netw Open. 2021 Jul 6;4(7):e2115687. doi: 10.1001/jamanetworkopen.2021.15687 (PMC8261607; doi:10.1001/jamanetworkopen.2021.15687)
Supplement: Supplement 3. — Data Sharing Statement [file jamanetwopen-e2115687-s003.pdf]

Data Sharing Statement

Winkelman. Feasibility and Acceptability of a Smoking Cessation Program for Individuals Released From an Urban, Pretrial Jail. *JAMA Netw Open*. Published July 06, 2021. doi:10.1001/jamanetworkopen.2021.15687

Data
Data available: No

Additional Information
Explanation for why data not available: This was a pilot randomized clinical trial with a final sample of N = 46. Given the small sample size and the importance of protecting the privacy of incarcerated populations, we cannot share these data and still maintain participants' privacy. We are able to share summary data not presented in the manuscript for purposes of meta-analyses and similar investigations.
